# The relationship between visceral fat thickness and bone mineral density in sedentary obese children and adolescents

**DOI:** 10.1186/1471-2431-13-37

**Published:** 2013-03-20

**Authors:** Ismael Forte Freitas Júnior, Jefferson Rosa Cardoso, Diego G Destro Christofaro, Jamile Sanches Codogno, Augusto César Ferreira de Moraes, Rômulo Araújo Fernandes

**Affiliations:** 1 Department of Physical Education, UNESP Univ Estadual Paulista, Presidente Prudente, Brazil; 2 Department of Physical Therapy, Universidade Estadual de Londrina - UEL, Londrina, Brazil; 3 Department of Physical Education, Universidade do Oeste Paulista, Presidente Prudente, Brazil; 4 Department of Physical Education, UNESP Univ Estadual Paulista, Rio Claro, Brazil; 5 Group of Scientific Research Related to Physical Activity. Department of Physical Education, UNESP Univ Estadual Paulista, Presidente Prudente, Brazil; 6 School of Medicine (FMUSP) of the Universidade de São Paulo, São Paulo, Brazil; 7 GENUD - Growth, Exercise, Nutrition and Development – Faculty of Health Sciences of the University of Zaragoza, Zaragoza, Spain; 8 GEPECIN - Research Group in Nutritional Sciences, Catholic Pontificate University/PR, Maringá, Brazil; 9 Universidade Estadual Paulista, Street Roberto Simonsen, 305, Presidente Prudente, SP, ZIP Code: 19060-900, Brazil

**Keywords:** Child, Adolescents, Obesity, Bone size, Bone density, Ultrasonography

## Abstract

**Background:**

Among adults, obesity has been positively related to bone mineral density. However, recent findings have pointed out that abdominal obesity could be negatively related to bone density. The above mentioned relationship is not clear among pediatric populations. Therefore, this cross-sectional study analyzed the relationship between thickness of abdominal adipose tissue and bone mineral variables in sedentary obese children and adolescents.

**Methods:**

One hundred and seventy five obese children and adolescents (83 male and 92 female) with ages ranging from 6 to 16 years-old were analyzed. Bone mineral content and density were estimated by dual-energy X-ray absorptiometry and ultrasound equipment which estimated the thickness of the abdominal adipose tissue. Pubertal stage was self-reported by the participants.

**Results:**

The mean age was 11.1 (SD = 2.6). Thickness of the abdominal adipose tissue was negatively related to bone mineral density (*r* = −0.17 [*r*_95%CI_: -0.03;-0.32]), independent of gender, pubertal stage and other confounders (β = −0.134 ± 0.042 [β_95%CI_: -0.217; -0.050]).

**Conclusions:**

In sedentary obese children and adolescents abdominal obesity is negatively related to bone mineral density, suggesting a potential link between abdominal obesity and osteoporosis.

## Background

In modern society osteoporosis is a highly occurring disease and constitutes a public health concern due to its impact on public costs [1]. Early life has been pointed out as a crucial period in the development of osteoporosis. Childhood and adolescence are phases of the human development during which the adult bone mass density is determined and, therefore, problems during this period of life could compromise bone health in adulthood [2].

Worldwide, children and adolescents are widely affected by obesity and its comorbidities [3-6]. Despite these related comorbidities, overweight/obesity has been associated with a lower occurrence of osteoporosis in adulthood. However, body weight is composed of lean and fat mass and the actual effect of the adipose tissue on bone mineral density (BMD) is not clear.

Moreover, the distribution of the adipose tissue could be a relevant confounder in this complex process that links obesity to osteoporosis. Recently, Bhupathiraju et al. [7] analyzing Porto Rican adults (47–79 years) observed that a higher abdominal fat mass (in kg) is related to a lower BMD, but the amount of visceral and subcutaneous abdominal adipose tissue were not assessed. Furthermore, there is an absence of data about this issue in pediatric populations. Understanding the relationship between pediatric obesity and bone health is relevant for health professionals [8-10], because childhood and adolescence are two critical periods in the prevention and development of diseases in adulthood (e.g. arterial hypertension, diabetes mellitus and dyslipidemias) [2,8-10]. So, the purpose of this study was to analyze the relationship between abdominal adipose tissue and BMD in obese children and adolescents.

## Methods

### Participants

The present study was approved by the Ethical Research Expert Committee of the Universidade Estadual Paulista in Presidente Prudente, Brazil (# 087/2008). The sample size was estimated through an equation for correlation’s coefficients, which took into consideration a power of 80% and significance of 5% (z = 1.96). A previous study analyzed the relationship between BMD and intra-abdominal adipose tissue (IAAT) and identified correlations ranging from *r* = −0.31 to *r* = −0.65 [11]. Thus, the lowest coefficient was inserted in the equation and the minimum sample size indicated was 80 subjects.

The subjects were invited, through television and newspaper advertising, to participate in this study. The participants contacted the researchers by phone and an appointment was made in order to take measurements at the Campus of the University. Initially, the diagnosis of obesity was based on body mass index cut-off points, adjusted by sex and age, developed by Cole et al. [12]. After a positive diagnosis of obesity, some inclusion criteria were also used to select the sample: i) aged between 6 and 17 years [chronological age computed taking into account their birthday and the measurement day]; ii) no engagement in regular physical activity within the three months prior to the study (established via a face-to-face interview with the child/adolescent and their parents); iii) a self-report of no diagnosis of either cardiovascular disease or regular medicine use and iv) a consent form signed by parents/guardians to participate in the study. Finally, one hundred and seventy five obese children and adolescents (83 male and 92 female) with ages ranging from 6 to 16 years-old were included in the study.

### Bone mineral density

Body composition and BMD were estimated by Dual-energy X-ray absorptiometry (DEXA) (Lunar DPX-NT; General Electric Healthcare, Little Chalfont, Buckinghamshire [software version 4.7]). The method estimated the percentage of body fat (%BF) and trunk fat mass (TFM [kg]), as well as, whole-body bone mineral density (BMD [g/cm^2^]). All data were collected by trained staff and all measurements were taken at the laboratory of the University, in a temperature controlled room. Each morning, before the first measurement, the DEXA equipment was calibrated by the same researcher according to the references provided by the manufacturer.

### Ultrasound measures of the abdominal adipose tissue

Ultrasound equipment (Toshiba Aplio Model Tochigi-ken, Japan) was used to measure the thickness of adipose tissue in the abdominal region. IAAT was defined as the distance between the internal face of the rectus abdominal muscle and the anterior wall of the aorta. Thus, the thickness (in cm) of IAAT was estimated. For statistical analysis, the values of IAAT were stratified into tertile (Tertile-1 [bottom]; middle Tertile-2 [middle]; Tertile-3 [top]). All measurements were taken by a trained physician, in a Hospital, in a room with a constantly controlled temperature. Each morning, before any measurements were taken, the device was calibrated and, according to the reference values provided by the manufacturer, the tests presented high reliability.

### Pubertal stage

Pubertal stage was self-assessed by the participants. The subjects received a standardized series of drawings to assess their own pubertal development (Girls: drawings with five stages of breast and female pubic hair development; Boys: drawings with five stages of genitalia and male pubic hair development) [13,14]. These scales have been previously validated in Brazilian pediatric populations [15,16]. The drawing had appropriate descriptions accompanying it. The results were placed by each subject in a locked box to guarantee the integrity and anonymity of the subjects, and only the main researcher had access to them. All pubertal measurements were performed at the University laboratory.

### Statistical analysis

The Kolmogorov-Smirnov (K-S) test, used to test the distribution of the numerical variables and logarithm transformation, was used in variables of non-parametric distribution. Mean, median, 95% confidence interval (95% CI), standard deviation (SD) and interquartile range (P_75_-P_25_) were used as descriptive statistics. For analysis of variance (ANOVA), homogeneity assumption was assessed in advance. If the assumption was in accordance, test F was performed and, in cases of statistical differences, the tertiles of IAAT were tested through Tukey’s multiple comparisons. Pearson´s correlation was used to analyze the relationship between the numerical variables. In cases of moderate-high correlations, multivariable models were elaborated using linear regression and, therefore, the correlations between BMD and IAAT were adjusted by potential confounders (sex, age, total body fatness, trunk fatness, height and pubertal stage). Chi-square test analyzed associations between categorical variables and Yates correction was applied in 2×2 contingence tables. Statistical significance was set at 5% and statistical software BioEstat version 5.0 was used for all analyses.

## Results

General characteristics of the analyzed sample are presented in Table 1. The K-S test indicated that most of the numerical variables were under non-parametric distribution, except for weight, height and %BF. Male gender made up 47.4% of the sample and there was a similarity between the numbers of boys and girls (*P* = 0.496). The mean age was 11.1 (SD = 2.6), and ranged from 6 to 16 years-old and the proportion of children and adolescents was similar (*P* = 0.112).

**Table 1:** General characteristics of obese children and adolescents (n = 175)

** Variables**	**Mean (SD)**	**(95% CI)**	**Median (P**_**75**_**-P**_**25**_**)**
Age (years)	11.1 (2.6)	(10.7; 11.5)	11 (4)
Weight (kg)	66.8 (19.2)	(63.9; 69.7)	64.1 (25.3)
Height (m)	1.50 (0.12)	(1.48; 1.52)	1.50 (0.20)
%BF	45.4 (5.2)	(44.6; 46.2)	45 (7.2)
TFM (kg)	13.8 (5.1)	(13.1; 14.5)	13.4 (6.3)
IAAT (cm)	4.35 (1.5)	(4.13; 4.58)	4.1 (1.8)
BMD (g/cm^2^)	1.04 (0.12)	(1.03; 1.06)	1.03 (0.19)
Categorical Variables	n (%)	*P*	
Gender		0.496	
Male	83 (47.4)		
Female	92 (52.6)		
Pubertal stage		0.037	
Stage 1	70 (40)		
Stages 2-3	62 (35.4)		
Stages 4-5	43 (24.6)		
Age		0.112	
6-10 years	77 (44)		
11-17 years	98 (56)		

SD = standard-deviation; 95%CI = 95% confidence interval; %BF = percentage of body fatness; Trunk-BF = trunk body fatness; IAAT = intra-abdominal adipose tissue; BMD = bone mineral density; TFM = trunk fat mass.

There were more children and adolescents at initial pubertal stage (40%) than final pubertal stage (24.6%) (*P* = 0.037). Children and adolescents at a higher pubertal stage had a higher BMD (Stage-1 = 0.96 ± 0.07 g/cm^2^; Stages 2-3 = 1.06 ± 0.10 g/cm^2^; Stages 4-5 = 1.16 ± 0.10 g/cm^2^; ANOVA *P* = 0.001). Boys and girls had similar values of BMD (1.05 ± 0.11 g/cm^2^ and 1.04 ± 0.12 g/cm^2^ [*P* = 0.630], respectively). Adolescents presented higher BMD than children (6-10 years = 0.95 ± 0.05 g/cm^2^ and 11-17 years = 1.12 ± 0.10 g/cm^2^ [*P* = 0.001], respectively).

**Figure 1:**
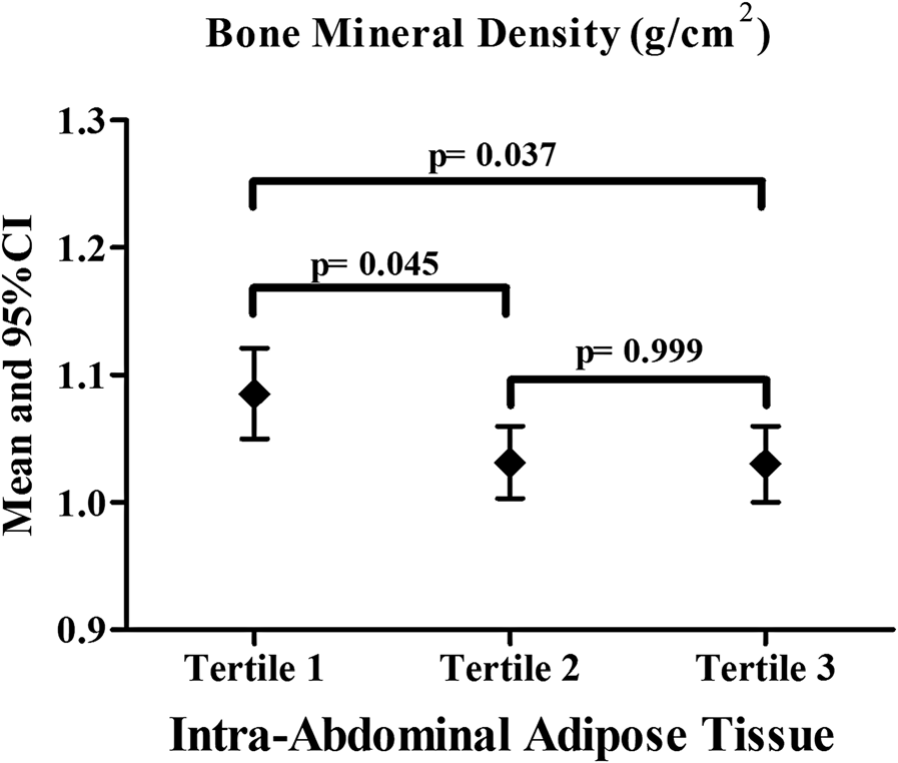
Bone mineral density according to intra-abdominal adipose tissue tertiles.

Age (*r* = 0.80), weight (*r* = 0.78), height (*r* = 0.70), TFM (*r* = 0.63) and pubertal stage (*r* = 0.66) were significantly related to BMD, except for %BF values (*r* = 0.09). In the multivariable model, IAAT was negatively related to BMD, independent of other confounders (Table 2). IAAT was stratified into tertiles and the values of BMD (Figure 1) were compared between them. Obese adolescents in the highest IAAT tertile presented lower BMD than those in the lowest IAAT tertile.

**Table 2:** Relationship between intra-abdominal adipose tissue and variables related to the bones of children and adolescents

	**Univariate**		**Multivariable model**	
	***r*(*r***_**95%CI**_**)**	**β**_**adjusted**_ **± SEM**	**(β**_**95%CI**_**)**	***P***
BMD*				
IAAT*	−0.17 (−0.03; -0.32)	−0.134 ± 0.042	(−0.217;-0.050)	0.002

* = numerical variable under logarithm transformation; β_adjusted_ = model adjusted by sex, age, height, total body fatness, trunk fatness and pubertal stage; BMD = bone mineral density; IAAT = intra-abdominal adipose tissue; SEM = standard error mean; 95%CI = 95% confidence interval.

## Discussion

A cross-sectional study, which identified that IAAT is inversely related to BMD. In this pediatric sample, the mean of IAAT was 4.35 cm which seems a normal value, because it was similar to another group of sedentary obese children and adolescents without non-alcoholic fat liver disease (mean value of 4.1 cm) [17]. On the other hand, the same group of obese children and adolescents had a significant decrease of 1.6 cm after one year of exercise intervention [17], supporting the relevance of the sedentary lifestyle as an inclusion criteria and evidencing the potential of prolonged engagement of physical exercise in combating comorbidities related to abdominal obesity [8,9]. The inclusion of the sedentary lifestyle was also important because there is a positive relationship between increased habitual physical activity and BMD [18].

Gender is an important variable related to skeletal formation. Male adolescents, more than girls, are affected by biological processes that accelerate bone development [2]. In the final stages of adolescence, boys are taller and have a higher bone mineral density [2]. Through childhood and adolescence, when compared to the female gender, boys have an increased likelihood of practicing sports [8,9], which are related to bone development. Moreover, during adulthood, hormonal characteristics of male obesity may exert deleterious effects on bone microarchitecture [19]. Therefore, the inclusion of gender as a potential confounder constitutes a methodological strength, because it indicates that IAAT is inversely related to BMD independent of this important confounder.

Agreeing with previous studies, in this sample there was a positive relationship between body weight/adiposity and BMD [11,20]. Indeed, obese subjects, from an early age, have increased bone density, mainly due to the stress occasioned by the increased weight on bone tissue that causes deformation and, hence, leads to bone remodeling [2]. Similarly, the same mechanical/biochemical process offers support to the idea that sports with impact are important tools in the promotion of adequate bone health in adolescents [2]. It is noteworthy that although obesity is positively related to BMD, more recent findings suggest that bone quality is compromised in obese subjects [21].

On the other hand, our findings point out that body fatness distribution should be considered as a potential confounder in this relationship, because increased IAAT was inversely related to BMD, independent of general obesity. Previous studies involving anthropometric (waist-to-hip ratio) [20] and DXA variables (abdominal fatness in kg) [7] identified similar relationship patterns in children and adults, respectively.

The inverse relationship between IAAT and BMD could be based on the action of adipokines produced by adipose tissue over growth mediators related to bone development. Nemet et al. [22] identified, in a longitudinal design, that the practice of very high intensity physical exercise simultaneously caused a significant increase in pro-inflammatory markers (tumor necrosis factor - alpha) and a subsequent decrease in growth mediators, such as insulin-like-growth-factor-I. Similarly, the adipose tissue located in the abdominal region (mainly the visceral one) has a special role in the release of adipokines into the bloodstream [23]. Therefore, it is possible to believe that IAAT could be a risk factor related to a harmful effect in bone remodeling and in turn to a risk factor of osteoporosis in adulthood.

Visceral adipose tissue is related to insulin resistance and insulin plays a role in the proliferation of osteoblasts. Thus, decreased insulin action may be one of the possible mechanisms by which obesity affects bone mass. In agreement, in a recent study, increased insulin concentration and HOMA-IR were considered negative predictors of bone mineral density in adolescents [24]. However, this is a recent finding that needs further top research focusing on the understanding of the physiological common mechanisms behind this association.

Our study has positive points, such as: (i) the sample size calculation; (ii) the use of adequate techniques to measure body composition/IAAT. On the other hand, limitations should be recognized: (i) the cross-sectional design (absence of causality statements), (ii) an absence of measurements relating to the intake of calcium and vitamin D and (iii) an absence of pro-inflammatory adipokines. Moreover, there were correlations of low magnitude between BMD and IAAT, indicating that other variables are important in this relationship and, therefore, further studies are necessary to identify them.

## Conclusions

In summary, our findings indicate that abdominal obesity negatively affects the bone density of obese children and adolescents, indicating that abdominal obesity could be a determinate in the development of osteoporosis in adulthood. Further studies should analyze whether this negative effect also occurs in non-obese youth.

## Abbreviations

BMD: Bone mineral density; IAAT: Intra-abdominal adipose tissue; BMI: Body mass index; TFM: Trunk fat mass; DEXA: Dual-energy X-ray absorptiometry; BF: Body fat; TFM: Trunk fat mass; K-S: Kolmogorov-smirnov; SD: Standard deviation; ANOVA: Analysis of variance; Cm: Centimeters; SEM: Standard-error mean.

## Authors’ contributions

IFFJ: (1) the conception and design of the study, acquisition of data and analysis and interpretation of data, (2) drafting the article and revising it critically for important intellectual content, (3) final approval of the version to be submitted. DGDC: (1) revising it critically for important intellectual content, (2) final approval of the version to be submitted. JRC: (1) revising it critically for important intellectual content, (2) final approval of the version to be submitted. ACFM: (1) revising it critically for important intellectual content, (2) final approval of the version to be submitted. JSC: (1) revising it critically for important intellectual content, (2) final approval of the version to be submitted. RAF: (1) the conception and design of the study (2) revising it critically for important intellectual content, (3) final approval of the version to be submitted. All authors read and approved the final manuscript.

## Competing interesting

The authors declare that there is no conflict of interest.

## Pre-publication history

The pre-publication history for this paper can be accessed here:

http://www.biomedcentral.com/1471-2431/13/37/prepub

## References

[B1] National Osteoporosis Foundation Strong voices for strong bones Washington, DC Available from: http://www.nof.org/files/nof/public/content/file/63/upload/49.pdf (cited 01 March 2013)

[B2] Malina RM Bouchard C Bar-Or O Growth, Maturation, and Physical Activity 2004 Human Kinetics: Champaign

[B3] Wang Y Monteiro C Popkin BM Trends of obesity and underweight in older children and adolescents in the United States, Brazil, China, and Russia Am J Clin Nutr 2002 75 971 977 12036801 10.1093/ajcn/75.6.971

[B4] Duncan S Duncan EK Fernandes RA Buonani C Bastos KD Segatto AF **Modifiable risk factors for overweight and obesity in children and adolescents from São Paulo. Brazil.** BMC Public Health 2011 11 585 10.1186/1471-2458-11-585 21781313 PMC3154175

[B5] Christofaro DG Ritti-Dias RM Fernandes RA Polito MD Andrade SM Cardoso JR High blood pressure detection in adolescents by clustering overall and abdominal adiposity markers Arq Bras Cardiol 2011 96 465 470 10.1590/S0066-782X2011005000050 21537530

[B6] Fernandes RA Freitas Júnior IF Codogno JS Christofaro DG Monteiro HL Roberto Lopes DM Resting heart rate is associated with blood pressure in male children and adolescents J Pediatr 2011 158 634 637 10.1016/j.jpeds.2010.10.007 21095617

[B7] Bhupathiraju SN Dawson-Hughes B Hannan MT Lichtenstein AH Tucker KL Centrally located body fat is associated with lower bone mineral density in older Puerto Rican adults Am J Clin Nutr 2011 94 1063 1070 10.3945/ajcn.111.016030 21865328 PMC3173024

[B8] Fernandes RA Christofaro DG Casonatto J Codogno JS Rodrigues EQ Cardoso ML Prevalence of dyslipidemia in individuals physically active during childhood, adolescence and adult age Arq Bras Cardiol 2011 97 317 323 10.1590/S0066-782X2011005000083 21830000

[B9] Fernandes RA Zanesco A Early physical activity promotes lower prevalence of chronic diseases in adulthood Hypertens Res 2010 33 8 926 931 20574424 10.1038/hr.2010.106

[B10] Sinaiko AR Donahue RP Jacobs DR Jr Prineas RJ Relation of weight and rate of increase in weight during childhood and adolescence to body size, blood pressure, fasting insulin, and lipids in young adults, The Minneapolis Children's Blood Pressure Study Circulation 1999 99 1471 1476 10.1161/01.CIR.99.11.1471 10086972

[B11] Do Prado WL De Piano A Lazaretti-Castro M De Mello MT Stella SG Tufik S Relationship between bone mineral density, leptin and insulin concentration in Brazilian obese adolescents J Bone Miner Metab 2009 27 613 619 10.1007/s00774-009-0082-6 19466592

[B12] Cole TJ Bellizzi MC Flegal KM Dietz WH Establishing a standard definition for child overweight and obesity worldwide: international survey BMJ 2000 320 1240 1243 10.1136/bmj.320.7244.1240 10797032 PMC27365

[B13] Marshall WA Tanner JM Variations in pattern of pubertal changes in girls Arch Dis Child 1969 44 291 303 10.1136/adc.44.235.291 5785179 PMC2020314

[B14] Marshall WA Tanner JM Variations in the pattern of pubertal changes in boys Arch Dis Child 1970 45 13 23 10.1136/adc.45.239.13 5440182 PMC2020414

[B15] Bojikianl LP Massa M Martini RH Teixeira CP Kissi MA Böhme MT F**emales' self-assessment of sexual maturation** Rev Bras Ativ Fis Saude 2002 7 24 34

[B16] Martin RH Uezu R Parra SA Arena SS Bojikian LP Böhme MT Males self-assessment of sexual maturation using drawings and photos Rev Paul Educ Fís 2001 15 212 222

[B17] Campos RM de Piano A da Silva PL Carnier J Sanches PL Corgosinho FC The role of pro/anti-inflammatory adipokines on bone metabolism in NAFLD obese adolescents: effects of long-term interdisciplinary therapy Endocrine 2012 42 146 156 10.1007/s12020-012-9613-3 22315014

[B18] Tobias JH Steer CD Mattocks CG Riddoch C Ness AR Habitual levels of physical activity influence bone mass in 11-year-old children from the United Kingdom: findings from a large population-based cohort J Bone Miner Res 2007 22 101 109 17014381 10.1359/jbmr.060913 PMC2742715

[B19] Bredella MA Lin E Gerweck AV Landa MG Thomas BJ Torriani M Bouxsein ML Miller KK Determinants of bone microarchitecture and mechanical properties in obese men J Clin Endocrinol Metab 2012 97 4115 4122 10.1210/jc.2012-2246 22933540 PMC3485587

[B20] Rhie YJ Lee KH Chung SC Kim HS Kim DH Effects of body composition, leptin, and adiponectin on bone mineral density in prepubertal girls J Korean Med Sci 2010 25 1187 1190 10.3346/jkms.2010.25.8.1187 20676331 PMC2908789

[B21] Shapses SA Sukumar D Bone metabolism in obesity and weight loss Annu Rev Nutr 2012 32 287 309 10.1146/annurev.nutr.012809.104655 22809104 PMC4016236

[B22] Nemet D Oh Y Kim HS Hill M Cooper DM Effect of intense exercise on inflammatory cytokines and growth mediators in adolescent boys Pediatrics 2002 110 681 689 10.1542/peds.110.4.681 12359780

[B23] Huang PL eNOS, metabolic syndrome and cardiovascular disease Trends Endocrinol Metab 2009 20 295 302 10.1016/j.tem.2009.03.005 19647446 PMC2731551

[B24] Campos RM Lazaretti-Castro M Mello MT Tock L Silva PL Corgosinho FC Influence of visceral and subcutaneous fat in bone mineral density of obese adolescents Arq Bras Endocrinol Metabol 2012 56 12 18 22460190 10.1590/s0004-27302012000100003

